# Use of a specialty endoscopy online platform for continuing medical education for clinical endoscopists during the COVID-19 pandemic

**DOI:** 10.1186/s12909-022-03516-2

**Published:** 2022-06-15

**Authors:** Guofu Li, Tingting Yu, Lichao Zhang, Haiming Du, Wei Zhang, Senlin Hou

**Affiliations:** The Second Hospital of HeBei Medical University, Shijiazhuang, People’s Republic of China

**Keywords:** Digestive endoscopy online platform, Clinical endoscopist, Continuing medical education

## Abstract

**Background:**

To explore the use of a digestive endoscopy professional online platform by domestic endoscopists and its application effect on endoscopists’ continuing medical education, analyse the related problems of continuing medical education using this method, and propose targeted improvement suggestions.

**Methods:**

Based on the “Doctor’s Circle” app, a questionnaire was sent to all members who successfully registered on the Hebei Biliary and Pancreatic Endoscopy Diagnosis and Treatment Alliance online platform. The questionnaire was available for 30 days. The questionnaire survey results were collected and counted for a grouping comparison.

**Results:**

By the deadline, 703 completed questionnaires had been received. After the registered doctors joined the platform, 469 (66.7%) experienced a significant influence on their own endoscopic operation ability level, and 354 (50.3%) felt a significant improvement in their ability to diagnose biliary- and pancreatic-related diseases. The application effect of the platform on members’ continuing medical education was affirmed by the vast majority of registered doctors. The clinical specialty of registered doctors, the length of time they joined the platform, the length of time they participated in the platform activities each time, and whether they played back course videos after the live broadcast of the course on the platform were the main factors affecting the application effect on continuing medical education (*P* < 0.05). Registered doctors who benefited significantly from the platform used it for 6–12 months, participated in activities for 1–2 hours each time, and often played back course videos.

**Conclusion:**

The new model of continuing medical education based on an online platform breaks through the constraints of traditional models and meets the individualized needs of every medical worker to improve their comprehension level. At present, the global outbreak of COVID-19 makes this learning mode increasingly popular among medical workers. We should constantly improve the organization of the content and methods of continuing medical education courses, make the online platform better serve the majority of medical workers, and effectively improve the comprehension levels of clinicians.

**Supplementary Information:**

The online version contains supplementary material available at 10.1186/s12909-022-03516-2.

## Background

“Internet Plus” is a new form of internet product. With the rapid development of the internet and information technology, the new ecological “Internet Plus” mode has brought about new developments in the medical field. A large number of reports have shown that “Internet Plus” is conducive to the development and sharing of medical resources [1, 2] and plays a vital role in improving the professional ability and comprehension quality of health professionals.

Continuing medical education (CME) is on-the-job education after completing medical school education and postgraduate education (graduate education and standardized resident training). CME, as the main form through which medical staff obtain advanced medical knowledge on theory and technology, plays an important role in promoting the integration of medical science and technology theory and clinical practice. Vigorous development of the content and form of continuing medical education is not only in line with the requirements of health development and the progress of medical science and technology but also the urgent need for every medical worker to improve their comprehension level. In today’s society, the popularity of smartphones also creates a new opportunity for medical staff to combine mobile medical information updates with clinical practice [3, 4]. In addition, with the development of the COVID-19 pandemic, various fields have been greatly affected, and the field of scientific research is also unable to escape the impact of the pandemic. Academic conferences at home and abroad were forced to cancel [5, 6]. Therefore, learning with the help of online platforms has also become the primary choice for the majority of medical workers.

Digestive endoscopy has become an important technical means for the diagnosis and treatment of digestive system diseases whose methods include gastroscopy, duodenoscopy, and colonoscopy and advanced diagnosis and treatment technologies, such as ERCP (endoscopic retrograde cholangiopancreatography), EUS (endoscopic ultrasonography), ESD (endoscopic submucosal dissection), and POEM (peroral endoscopic myotomy). In recent years, with the rapid development of internet technology, online platforms on digestive endoscopy have undergone unprecedented changes. A large number of widely used online platforms have emerged. Based on the “Doctor’s Circle” (a mobile app called the mobile partner of doctors), the “Hebei Biliary and Pancreatic Endoscopy Diagnosis and Treatment Alliance” is mainly a platform for the professional exchange of information on biliary and pancreatic endoscopy, such as information on biliary- and pancreatic-related diseases, ERCP and ultrasonic endoscopy. The members of this learning platform are mainly composed of professionals engaged in biliary- and pancreatic-related diseases, gastroenterology, general surgery and digestive endoscopy. It has been established and developed since 2014. The platform includes cutting-edge medical information, clinical experience sharing, academic conference video sharing and difficult cases discussion, and regularly invites domestic well-known digestive endoscopy experts to give lectures through webcast. This platform is an independent learning platform. The basic purpose of this platform is to help digestive endoscopy clinicians and clinicians of other specialties freely carry out continuing education, continuously improve the skill levels and academic levels of clinical endoscopists in China, and encourage experts in the field and even beginners in endoscopy to interact and share their experiences, mutual supervision and common progress.

To date, there is no research on endoscopists’ use of professional digestive endoscopy online platforms and the impact of these on endoscopists’ continuing education at home and abroad. This study will fill the gap in this research.

## Methods

### Respondents

The respondents were successfully registered as clinicians on the Hebei Biliary and Pancreatic Endoscopy Diagnosis and Treatment Alliance online platform. These respondents come from all over the country and have successfully registered as members of this learning platform. In their clinical work, they are mainly engaged in gastroenterology, general surgery and digestive endoscopy, and even include young graduate students and professional nurses who cooperate in the process of endoscopic surgery. Some of them are already experts in digestive endoscopy, and some are young scholars who have just come into contact with digestive endoscopy.

### Research methods

Based on the “Doctor’s Circle” app, a questionnaire was sent to all members (3938 at the time the questionnaire was issued) who were successfully registered on the Hebei Biliary and Pancreatic Endoscopy Diagnosis and Treatment Alliance online platform, and the questionnaire survey results were collected. After consulting the relevant literature and experts in relevant fields and combining those results with the research purpose of this study, the “Questionnaire on the Participation of Registered Doctors of the Hebei Biliary and Pancreatic Endoscopy Diagnosis and Treatment Alliance” was designed and prepared by the “SOJUMP” app.

The “SOJUMP” app we use is a professional platform for online questionnaire survey, evaluation and voting. It can provide personalized online questionnaire design, support for classification and statistics function, download data and original answers for free, and support the functions of mobile phone filling and “WeChat” mass sending.

The “Doctor’s Circle” is an application tool tailored for doctors. It is a real-name doctor community. It contains a professional academic exchange circle led by many experts. It is a professional medical database and an academic carrier for innovative knowledge sharing and interactive exchange. This learning platform is based on application tools such as “Doctor’s Circle”.

The contents of the questionnaire included the following: 1) The basic information of the respondents, including sex, age, current highest educational level, hospital level, specialty and professional title used in clinical work; 2) the learning situation of the respondents who logged on to the platform, including login frequency, time since joining, time spent participating in platform activities each time, playing back course videos, etc.; 3) the effect of the platform on the continuing medical education of registered doctors, including the impact on their own endoscopic operation ability level and the diagnosis and treatment of related biliary and pancreatic diseases; 4) the evaluation of the platform; and 5) shortcomings and suggestions for improvement. This questionnaire could be accessed by using the SOJUMP QR code poster or directly clicking the provided network link. First, the questionnaire briefly introduced the purpose and instructions of the survey to the participants. In the questionnaire, we informed the respondents of various precautions, obtained the informed consent of the respondents, and promised to keep the contents of the questionnaire strictly confidential. Its content consisted of 16 single-choice questions, 4 multiple-choice questions and 1 voluntary short-answer question. The answering method was convenient, and it took approximately 2 ~ 3 min to complete all the questions. To ensure the accuracy and authenticity of the questionnaire results, we restricted the IP address and WeChat responses. Furthermore, to shorten the survey cycle, the questionnaire was available for 30 days.

### Statistical methods

The questionnaire data were collected through the “SOJUMP” system and statistically analysed by SPSS 23 statistical software. Count data are expressed as a rate or constituent ratio, and a difference between the two population rates or constituent ratios was inferred. The chi-square test or Fisher’s exact test was used, with *P* < 0.05 indicating a statistically significant difference. The constituent ratios of multiple samples were compared by the R × C diagram chi-square test, and the chi-square segmentation method was used for multiple comparisons among multiple sample rates.

## Results

### General information

Up to the deadline, 703 completed questionnaires were received. Finally, 703 valid research questionnaires were selected, with an effective rate of 100%. From the data collected from the questionnaire, there were 521 male participants (71.1%) and 182 female participants (25.8%); the age distribution was 39 people under the age of 19 (5.5%), 359 people aged 20 ~ 29 (51.0%), 266 people aged 30 ~ 39 (37.8%), 36 people aged 40 ~ 49 (5.1%), and 3 people over 50 (0.4%); the education level distribution was 169 with an associate’s degree (24.0%), 439 with an undergraduate degree (62.6%), and 95 (13.5%) with a master’s degree or above; hospital grade distribution was 175 (24.8%) class 3A hospitals, 232 (33%) class 3B hospitals, 226 (32.1%) class 2A hospitals, 51 (7.2%) class 2B hospitals, and 19 (2.7%) other-class hospitals; the professional distribution of clinical work was 146 (20.7%) clinicians in gastroenterology, 276 (39.2%) in general surgery, 213 (30.3%) in endoscopy centres, 51 (7.2%) in nursing and 17 (2.4%) in other facilities or specialties; and the title distribution was 108 medical students (15.3%), 180 residents (25.6%), 226 attending doctors (32.1%), 118 deputy chief doctors (16.7%), 42 chief doctors (5.9%), and 29 other titles/identities not listed (4.1%). The distribution of endoscopic operation types (multiple choices) included ERCP, which accounted for 48.6% (342/703), EUS, which accounted for 52.9% (372/703), gastroscopy and colonoscopy, which accounted for 57.0% (401/703), ESD, which accounted for 31.2% (220/703) and others, which accounted for 5.6% (40/703).

### Statistics on the cumulative number of ERCP cases completed by individual registered doctors (Fig. 1) and affiliated hospitals (Fig. 2)

**Fig. 1:**
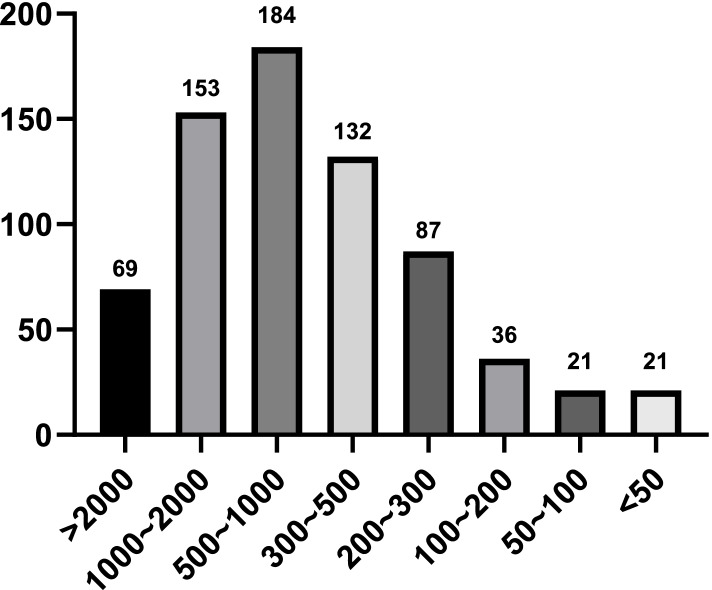
Number of cumulative ERCP cases completed by registered physicians

**Fig. 2:**
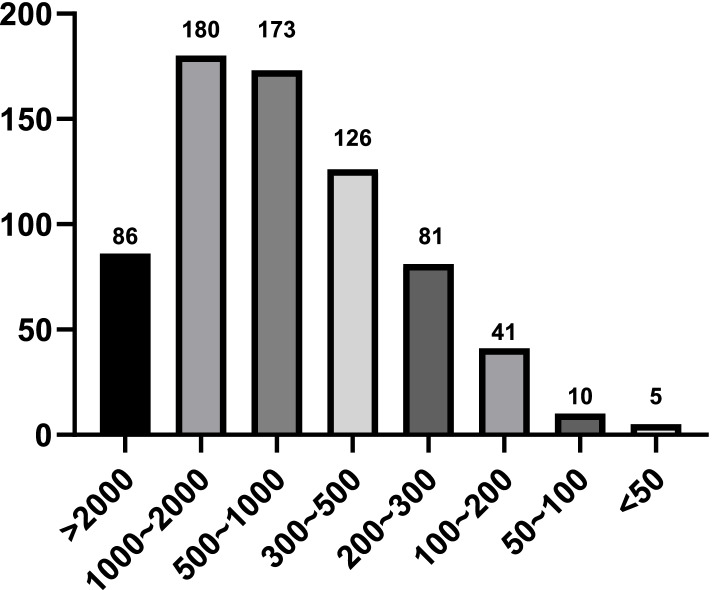
Number of cumulative ERCP cases completed by the hospitals of registered physicians

### Application effect of the Hebei biliary and pancreatic endoscopy diagnosis and treatment alliance platform on continuing medical education for registered doctors

#### Learning situation of registered doctors on the Hebei biliary and pancreatic endoscopy diagnosis and treatment alliance platform (hereinafter referred to as “the platform”)

The frequency distribution of registered doctors accessing the platform was as follows: 105 (14.9%) once a week or less, 286 (40.6%) twice to three times a week, 228 (32.43%) four to five times a week, 63 (8.9%) six to seven times a week and 21 (2.9%) more than seven times a week. The length of time that registered doctors joined the platform was as follows: 136 (19.3%) joined for less than 6 months, 328 (46.6%) for 6–12 months, 169 (24.0%) for 1–2 years and 70 (9.9%) for more than 2 years. The distribution of the main activities of registered doctors on the platform (multiple choices) was as follows: 38.8% (273/703) searched for information or services needed on the platform, 47.5% (334/703) simply browsed posts or accessed content, 52.0% (366/703) found new posts and provided help, 45.3% (319/703) posted and sought information or services, 33.2% (234/703) interacted with friends and exchanged information, and 17.0% (120/703) interacted with teaching experts and solved problems online. The time that registered doctors spent participating in platform activities each time was as follows: 77 (10.9%) spent less than half an hour participating, 380 (54.0%) spent half an hour to one hour participating, 214 (30.4%) spent one to two hours participating and 32 (4.5%) spent more than two hours participating. After the live broadcast of the course on the platform, 341 people (48.5%) watched the playback often, 276 people (39.2%) occasionally watched the playback, and 86 people (12.2%) did not watch the playback. After the registered doctors joined the platform, the impact on their own endoscopic operation ability level was as follows: 469 (66.7%) reported a significant effect, 209 (29.7%) reported a general effect, and 25 (3.5%) reported no effect. After joining the platform, the specific improvement in endoscopic operation skills (multiple choices) included skilfully inserting the endoscope into the designated position, which accounted for 51.4% (362/703), intubation of the bile duct or pancreatic duct, which accounted for 50.9% (358/703), removal of bile duct stones and pancreatic duct stones, which accounted for 53.0% (373/703), treatment of complications after endoscopic surgery, which accounted for 44.8% (315/703), diagnosis under an endoscopic ultrasound, which accounted for 22.3% (157/703), and other skills, which accounted for 1.7% (12/703). Regarding the impact on their diagnostic abilities of biliary- and pancreatic-related diseases, 354 (50.3%) reported a significant improvement, 162 (23.0%) reported no significant improvement, 130 (18.4%) reported no improvement, 49 (6.9%) reported decreased diagnostic ability and 8 (1.1%) were unsure.

#### Comparison of the influence of the platform on the endoscopic operation ability levels of registered doctors under different conditions

There was no statistically significant difference in the influence of sex, age, educational background, hospital grade or professional title of registered doctors on their own endoscopy ability level since they joined the platform (*P* > 0.05). There was a statistically significant difference in the influence of the specialty of registered doctors engaged in clinical work on their own endoscopy ability level (*P* < 0.05) (see Table 1). After further analysis, the registered doctors in endoscopy departments experienced the most significant effect on the improvement in endoscopy ability level. The reason for this finding is that the platform is mainly a platform for professional communication on digestive endoscopy dominated by ERCP and ultrasonic endoscopy. Endoscopic centres are more engaged in such operations than gastroenterology and general surgery departments.

**Table 1:** The effect of different situations on registered physicians’ endoscopic operation ability level after they joined the platform was compared (n, %)

Project	Influence of registered doctors’ endoscopic operation ability level after joining the platform			χ2	*P*
	Significant effect	Average effect	No effect		
Sex				5.387	0.068
Male	337 (64.6)	167 (32.0)	17 (3.2)		
Female	132 (72.5)	42 (23.0)	8 (4.3)		
Age				11.872	0.131
Under 19	22 (56.4)	15 (38.4)	2 (5.1)		
20 ~ 29 years old	229 (63.7)	116 (32.3)	14 (3.8)		
30 ~ 39 years old	189 (71.0)	71 (26.6)	6 (2.2)		
40 ~ 49 years old	26 (72.2)	7 (19.4)	3 (8.3)		
Over 50	3 (100)	0 (0)	0 (0)		
Education				1.163	0.884
Associate’s degree	110 (65.0)	54 (1.9)	5 (2.9)		
Undergraduate degree	298 (67.8)	125 (28.4)	16 (3.6)		
Master’s degree or above	61 (64.2)	30 (31.5)	4 (4.2)		
Hospital grade				14.639	0.055
Class 3A	127 (72.5)	43 (24.5)	5 (2.8)		
Class 3B	147 (63.3)	80 (3.4)	5 (2.1)		
Class 2A	154 (68.1)	62 (27.4)	10 (4.4)		
Class 2B	32 (62.7)	17 (33.3)	2 (3.9)		
Other	9 (47.3)	7 (36.8)	3 (15.7)		
Specialty of clinical work				20.224	0.007
Gastroenterology	115 (78.7)	30 (20.5)	1 (0.6)		
General surgery	183 (66.3)	84 (30.4)	9 (3.2)		
Endoscopy	128 (60.0)	75 (35.2)	10 (4.6)		
Nursing specialty	33 (64.7)	15 (29.4)	3 (5.8)		
Other	10 (58.8)	5 (29.4)	2 (11.7)		
Title				16.472	0.07
Medical student	80 (74.0)	25 (23.1)	3 (2.7)		
Resident	125 (69.4)	48 (26.6)	7 (3.8)		
Attending doctor	155 (68.5)	67 (29.6)	4 (1.7)		
Deputy chief physician	66 (55.9)	46 (38.9)	6 (5.0)		
Chief physician	26 (61.9)	14 (33.3)	2 (4.7)		
Other titles/identities not listed	17 (58.6)	9 (31.0)	3 (10.3)		

#### The influence of the platform on registered doctors’ ability to diagnose biliary- and pancreatic-related diseases in different situations was compared

There was no significant difference in the influence of age on the ability to diagnose biliary- and pancreatic-related diseases (*P* > 0.05). There was a significant difference in the influence of sex, educational background, hospital grade, specialty and professional title on the physicians’ ability to diagnose biliary- and pancreatic-related diseases (*P* < 0.05) (see Table 2). Further statistical analysis showed that male clinicians working in class 3A hospitals and engaged in general surgery significantly improved their ability to diagnose biliary- and pancreatic-related diseases, which is closely related to the fact that most clinical doctors who are engaged in general surgery are men. In addition, clinical medical students, including professional postgraduates and those training in public clinics, have been struggling in their jobs for some time, but there is still a large gap in disease diagnosis, treatment and clinical experience compared with other senior doctors. After the “charging” of continuous medical education, the learning effect was significantly improved. Moreover,we found that by learning on this platform, graduate students and residents have improved their understanding of the professional field of digestive endoscopy and their ability to diagnose and treat biliary- and pancreatic-related diseases. For nurses, it has played a great role in the cooperation of endoscopic surgery and postoperative nursing.

**Table 2:** Comparison of the influence of the platform on different registered doctors’ ability to diagnose biliary and pancreatic diseases (n, %)

Project	Influence on registered doctors’ ability to diagnose biliary and pancreatic diseases after joining the platform					χ2	*P*
	Significantly improved	The improvement was not obvious	Not improved	Lower than before	Unclear		
Sex						22.195	0.000
Male	238 (45.6)	131 (25.1)	109 (20.9)	39 (7.4)	4 (0.7)		
Female	116 (63.7)	31 (17.0)	21 (11.5)	10 (5.4)	4 (2.1)		
Age						9.821	0.904
Under 19	16 (41.0)	10 (25.6)	11 (28.2)	2 (5.1)	0 (0.0)		
20 ~ 29 years old	177 (49.3)	82 (22.8)	69 (19.2)	26 (7.2)	5 (1.3)		
30 ~ 39 years old	137 (51.5)	63 (23.6)	45 (16.9)	19 (7.1)	2 (0.7)		
40 ~ 49 years old	21 (58.3)	7 (19.4)	5 (13.8)	2 (5.5)	1 (2.7)		
Over 50	3 (100.0)	0 (0.0)	0 (0.0)	0 (0.0)	0 (0.0)		
Education						17.341	0.027
Associate’s degree	92 (54.4)	33 (19.5)	29 (17.1)	9 (5.3)	6 (3.5)		
Undergraduate degree	211 (48.0)	105 (23.9)	88 (20.0)	34 (7.7)	1 (0.2)		
Master’s degree or above	51 (53.6)	24 (25.2)	13 (13.6)	6 (6.3)	1 (1.0)		
Hospital grade						35.302	0.002
Class 3A	112 (64.0)	36 (20.5)	18 (10.2)	7 (4.0)	2 (1.1)		
Class 3B	102 (43.9)	61 (26.2)	55 (23.7)	13 (5.6)	1 (0.4)		
Class 2A	107 (47.3)	49 (21.6)	44 (19.4)	23 (10.1)	3 (1.3)		
Class 2B	23 (45.0)	11 (21.5)	12 (23.5)	4 (7.8)	1 (1.9)		
Other	10 (52.6)	5 (26.3)	1 (5.2)	2 (10.5)	1 (5.2)		
Specialty of clinical work						35.3	0.002
Gastroenterology	98 (67.1)	24 (16.4)	17 (11.6)	7 (4.7)	0 (0.0)		
General surgery	123 (44.5)	77 (27.8)	56 (20.2)	18 (6.5)	2 (0.7)		
Endoscopy	99 (46.4)	47 (22.0)	45 (21.1)	19 (8.9)	3 (1.4)		
Nursing specialty	27 (52.9)	10 (19.6)	10 (19.6)	2 (3.9)	2 (3.9)		
Other	7 (41.1)	4 (23.5)	2 (11.7)	3 (17.6)	1 (5.8)		
Title						41.321	0.001
Medical student	66 (61.1)	22 (20.3)	14 (12.9)	6 (5.5)	0 (0.0)		
Resident	97 (53.8)	38 (21.1)	37 (20.5)	7 (3.8)	1 (0.5)		
Attending doctor	106 (46.9)	56 (24.7)	48 (21.2)	14 (6.1)	2 (0.8)		
Deputy chief physician	47 (39.8)	32 (27.1)	23 (19.4)	16 (13.5)	0 (0.0)		
Chief physician	24 (57.1)	8 (19.0)	6 (14.2)	3 (7.1)	1 (2.3)		
Other titles/identities not listed	14 (48.2)	6 (20.6)	2 (6.8)	3 (10.3)	4 (13.7)		

#### Comparison of the influence of different registered doctors’ learning situations on their endoscopic operation ability level after joining the platform

There was no significant difference in the influence of the frequency of registered doctors logging on to the platform on their own endoscopic operation ability level (*P* > 0.05). However, there was a significant difference in the influence of the length of time registered doctors were on the platform, the time they participated in platform activities each time, and whether they watched the playback of the course videos after the live broadcast on the platform on their own endoscopic operation ability level (*P* < 0.05) (see Table 3).

**Table 3:** Comparison of the influence of different registered doctors’ learning situations on their own endoscopic operation ability level after they joined the platform (n, %)

Project	After joining the platform, registered doctors’ endoscopic operation ability levels were affected			χ2	P
	Significant effect	Average effect	No effect		
Frequency of platform access				6.448	0.570
Once a week or less	71 (67.6)	31 (29.5)	3 (2.8)		
2 ~ 3 times a week	196 (68.5)	80 (27.9)	10 (3.4)		
4 ~ 5 times a week	151 (66.2)	70 (30.7)	7 (3.0)		
6 ~ 7 times a week	35 (55.5)	24 (38.0)	4 (6.3)		
More than 7 times	16 (76.1)	4 (19.0)	1 (4.7)		
Length of time on the platform				19.855	0.003
Less than 6 months	97 (71.3)	35 (25.7)	4 (2.9)		
6 ~ 12 months	224 (68.2)	94 (28.6)	10 (3.0)		
1 ~ 2 years	92 (56.8)	69 (40.8)	8 (4.7)		
More than 2 years	56 (80.0)	11 (15.7)	3 (4.2)		
Time spent in each platform activity				16.101	0.013
Less than half an hour	59 (76.6)	13 (16.8)	5 (6.4)		
Half an hour to an hour	259 (68.1)	111 (29.2)	10 (2.6)		
One to two hours	127 (59.3)	79 (36.9)	8 (3.7)		
More than two hours	24 (75.0)	6 (18.7)	2 (6.2)		
Played back course videos				123.368	0
Watched often	296 (86.8)	39 (11.4)	6 (1.7)		
Occasionally	134 (48.5)	130 (47.1)	12 (4.3)		
Did not watch playback	39 (45.3)	40 (46.5)	7 (8.1)		

#### Comparison of the influence of different registered doctors’ learning situations on their ability to diagnose biliary- and pancreatic-related diseases after joining the platform

The influence of the frequency with which registered doctors logged on to the platform, the duration of time on the platform, the time spent participating in platform activities each time, and whether the course videos were played back after the live broadcast of the course on the ability to diagnose biliary- and pancreatic-related diseases were significant (*P* < 0.05) (see Table 4).

**Table 4:** Comparison of the influence of different registered doctors’ learning situations on the diagnosis of biliary and pancreatic diseases after they joined the platform (n, %)

Project	Influence of registered doctors’ learning situations on the diagnosis of biliary and pancreatic diseases after they joined the platform					χ2	P
	Significantly improved	The improvement was not obvious	Not improved	Lower than before	Unclear		
Frequency of platform access						25.678	0.038
Once a week or less	66 (62.8)	17 (16.1)	13 (12.3)	7 (6.6)	2 (1.9)		
2 ~ 3 times a week	148 (51.7)	64 (22.3)	50 (17.4)	20 (6.9)	4 (1.3)		
4 ~ 5 times a week	101 (44.2)	63 (27.6)	50 (21.9)	14 (6.1)	0 (0.0)		
6 ~ 7 times a week	26 (41.2)	16 (25.3)	14 (22.2)	6 (9.5)	1 (1.5)		
More than 7 times	13 (61.9)	2 (9.5)	3 (14.2)	2 (9.5)	1 (4.7)		
Length of time on the platform						53.191	0.000
Less than 6 months	75 (55.1)	28 (20.5)	22 (16.1)	7 (5.1)	4 (2.9)		
6 ~ 12 months	161 (49.0)	85 (25.9)	59 (17.9)	21 (6.4)	2 (0.6)		
1 ~ 2 years	62 (36.6)	41 (24.2)	47 (27.8)	18 (10.6)	1 (0.5)		
More than 2 years	56 (80.0)	8 (11.4)	2 (2.8)	3 (4.2)	1 (1.4)		
Time spent on each platform activity						25.133	0.011
Less than half an hour	44 (57.1)	19 (24.6)	7 (9.0)	3 (3.8)	4 (5.1)		
Half an hour to an hour	192 (50.5)	91 (23.9)	74 (19.4)	21 (5.5)	2 (0.5)		
One to two hours	100 (46.7)	43 (20.0)	47 (21.9)	22 (10.2)	2 (0.9)		
More than two hours	18 (56.2)	9 (28.1)	2 (6.2)	3 (9.3)	0 (0.0)		
Played back the course videos						199.519	0.000
Watched often	260 (76.2)	33 (9.6)	35 (10.2)	13 (3.8)	0 (0.0)		
Occasionally	73 (26.4)	100 (36.2)	74 (26.8)	27 (9.7)	2 (0.7)		
Did not watch playback	21 (24.4)	29 (33.7)	21 (24.4)	9 (10.4)	6 (6.9)		

Regarding the statistical analysis of the learning situation of registered doctors accessing the platform, there was no significant difference in the influence of the frequency of registered doctors accessing the platform on their own endoscopic operation ability level (*P* > 0.05), but the length of time that registered doctors joined the platform, the time spent participating in platform activities each time and playing back the videos after the live broadcast of the course had a significant influence on their endoscopic operation ability level (*P* < 0.05). Further statistical analysis showed that the length of time that registered doctors joined the platform or the time they spent participating in activities did not indicate that more time spent on the platform produced a better endoscopic operation ability level in the doctors. In contrast, the registered doctors who joined the platform for 6–12 months and the registered doctors who participated in platform activities for one to two hours each time benefited significantly. In addition, the frequency with which registered doctors logged on to the platform, the length of time they joined the platform, the time they participated in platform activities each time, and whether they watched the playback of the course after the live broadcast had a statistically significant difference on the doctors’ abilities to diagnose biliary- and pancreatic-related diseases (*P* < 0.05). It is worth mentioning that the latter (the influence on the ability to diagnose biliary- and pancreatic-related diseases) was compared with the former (influence on the endoscopic operation ability level), and there were differences in the frequency with which registered doctors accessed the platform. The students who logged on to the platform 4–5 times a week for learning significantly improved their ability to diagnose a disease. The reason is that for theoretical knowledge, medical workers can remember and master the lessons by repeatedly logging on to the platform for review. However, to thoroughly master actual clinical surgery skills, this alone is far from enough. Practice makes perfect only through repeated practice, which is also a limitation of continuing medical education based on online platforms.

### Evaluation of registered doctors on the Hebei biliary and pancreatic endoscopy diagnosis and treatment alliance platform

A total of 59.3% (417/703) of participants thought that the platform had a large number of knowledgeable resources, with wide coverage and high reliability. A total of 69.8% (491/703) thought that the platform had a strong and friendly academic atmosphere and that everyone was willing to engage in communication to exchange and increase knowledge and share experiences. A total of 45.3% (319/703) thought that the platform had a reasonable, user-friendly design and perfect functions. They believed that the platform relied on a strong medical team that was very friendly and had a close relationship with users. A total of 48.0% (338/703) of the team members trusted the platform very much; 94.0% (661/703) of registered doctors were very willing to recommend this platform to others, and 5.9% (42/703) were unwilling to recommend this platform to others. The application effect of the platform on members’ continuing medical education was affirmed by the vast majority of registered doctors.

## Discussion

In recent years, with the rapid development of internet technology, digestive endoscopy online platforms have undergone unprecedented changes. A large number of widely used online platforms have emerged. The Hebei Biliary and Pancreatic Endoscopy Diagnosis and Treatment Alliance based on the “Doctor’s Circle” app is one of them. This platform is mainly a platform for the professional exchange of information on biliary and pancreatic endoscopy, such as information on biliary- and pancreatic-related diseases, ERCP and ultrasonic endoscopy. The basic purpose of this platform is to help digestive endoscopy clinicians and clinicians of other specialties freely carry out continuing education, continuously improve the skill levels and academic levels of clinical endoscopists in China, and encourage experts in the field and even beginners in endoscopy to interact and share their experiences, mutual supervision and common progress. In recent years, domestic studies [7] have confirmed that an orthopaedic professional online platform is conducive to improving the skill levels and clinical levels of orthopaedic clinicians in China. To date, there is no research on the use of professional digestive endoscopy online platforms by endoscopists at home or abroad or the impact of professional digestive endoscopy online platforms on the continuing education of endoscopists. This study filled the gap in this research by means of a questionnaire.

According to the results of this survey, 69.8% (491/703) of registered doctors thought that the platform had a strong and friendly academic atmosphere and that everyone was willing to engage in communication to exchange and increase their knowledge and share experiences. In total, 59.3% (417/703) of registered doctors thought that the platform had a large number of knowledgeable resources, with wide coverage and high reliability. In total, 52.0% (366/703) of registered doctors most enjoyed finding new posts and providing help, 47.5% (334/703) most enjoyed simply browsing posts or accessing content, and 45.3% (319/703) most enjoyed posting and seeking information or services. The platform provides rich medical information resources for medical workers with heavy daily workloads and a convenient and fast way to obtain medical knowledge and resolve doubts, breaking through the constraints of time, place and funds. The establishment of a platform with good communication methods also promotes real-time interactive learning, which is conducive to the sharing of medical education resources and the improvement in medical skills. At present, the COVID-19 pandemic is severe, and the academic field of science and technology has been greatly affected. Various academic conferences at home and abroad were cancelled. Online platforms have become the primary choice of learning for the majority of medical workers. Previous studies have confirmed that surgical online platforms are helpful to improve the surgical training of surgeons [8, 9]. Cutting-edge medical information, diagnosis and treatment experience sharing, conference videos of the Digestive Endoscopy Society, surgical videos on ERCP and EUS and so on covered by the platform are conducive to improving the clinical and academic skills and comprehension quality levels of clinical endoscopists and meeting the needs of individual development. However, it should be noted that the platform rarely includes information on the humanities and social sciences. It is hoped that the person in charge of the platform can add relevant knowledge to meet the requirements of medical talent training combining social disciplines and humanistic qualities.

The limitations of this study are as follows: at present, the number of people successfully registered on the Hebei Biliary and Pancreatic Endoscopy Diagnosis and Treatment Alliance online platform is 3938. Due to the short duration of the questionnaire (30 days from beginning to end), only 703 completed questionnaires were received, and the amount of data is relatively small. When designing the questionnaire, we took into account as many relevant factors as possible, including the basic information of the respondents such as sex, age, educational background and clinical specialty, as well as their learning condition such as the frequency of accessing the learning platform and the length of joining the platform. However, it is difficult to involve all the factors related to it. For example, there may also be statistically significant differences in the learning contents that members focus on on this platform. Moreover, members can also read relevant professional books and literature by themselves, or participate in training courses and other ways to improve their ability level. In this regard, this study has limitations and needs to be improved in the future. In addition, this study uses the questionnaire survey method to collect data. The results are obviously affected by the subjective factors of the respondents and lack objective evaluation criteria, and the results may have some bias, prompting a need for an improved research design in the future.

## Conclusions

The new continuing medical education model based on online platforms surpasses the constraints of traditional models, enables medical personnel to obtain cutting-edge medical science and technology information without restrictions on time, place and funds, meets the individualized needs of each medical worker to improve their comprehension level, and achieves the purpose of continuing medical education. At present, the COVID-19 pandemic makes this learning method increasingly popular among medical workers. Relevant departments should pay attention to the development and utilization of online platforms, improve the organization of the content and form of continuing medical education courses, and establish and improve the supervision and control system and feedback mechanism to make online platforms better serve the majority of medical workers and effectively improve the comprehension levels of clinicians.

## Supplementary Information

**Additional file 1.**

## Data Availability

All data generated and/or analysed during this study are included in this published article. The data used and/or analysed during the current study are available from the corresponding author upon reasonable request.

## Abbreviations

CME: Continuing medical education
ERCP: Endoscopic retrograde cholangiopancreatography
EUS: Endoscopic ultrasonography
ESD: Endoscopic submucosal dissection
POEM: Peroral endoscopic myotomy
